# Synergistic Effects of Bismuth Thiols and Various Antibiotics Against *Pseudomonas aeruginosa* Biofilm

**DOI:** 10.5812/jjm.9142

**Published:** 2014-03-01

**Authors:** Maryam Varposhti, Ahya Abdi Ali, Parisa Mohammadi

**Affiliations:** 1 Department of Biology, Faculty of Sciences, Alzahra University, Tehran, IR Iran

**Keywords:** Biofilms, *Pseudomonas aeruginosa*, Antibacterial Agents

## Abstract

**Background:**

*Pseudomonas aeruginosa* is an opportunistic pathogen that takes advantages of some weaknesses in the immune system to initiate an infection. Biofilms of *P. aeruginosa* can cause chronic opportunistic infections in immunocompromised and elderly patients. This bacterium is considered as a model organism to study antibiotic resistance as well as biofilm formation. In the biofilm structures, bacteria are protected from many harmful environmental factors such as fluctuations in the level of oxygen and nutrients, and the alterations of pH as well as sensitivity to antibiotics. Decreased permeability of biofilms is one of the important reasons of antimicrobial resistance in bacteria.

**Objectives:**

In this study the anti-biofilm activity of bismuth thiols in combination with ciprofloxacin, imipenem and ceftazidime against the *P. aeruginosa* biofilm was investigated.

**Materials and Methods:**

Checkerboard method was used to test the susceptibility of biofilms against various antimicrobial combinations. The biofilm formation was measured by 2,3-bis (2-methoxy-4-nitro-5-sulfo-phenyl)-2H-tetrazolium-5-carboxanilide (XTT) colorimetric assay. The fractional bio-film inhibitory concentration was reported for each agent.

**Results:**

The combination of bismuth ethanedithiol with ciprofloxacin showed synergistic inhibitory effect on the *P. aeruginosa* biofilm formation. The combination of bismuth ethanedithiol ciprofloxacin, ceftazidime and imipenem showed synergistic inhibitory effects on the biofilm formation. Furthermore, the combination of bismuth ethanedithiol, imipenem and ceftazidime did not show any synergistic inhibitory effect on biofilm formation.

**Conclusions:**

Our studies show that using appropriate concentrations of bismuth thiols in combination with various antibiotics can act synergistically against *P. aeruginosa* biofilm formation.

## 1. Background

Biofilms are specific structures of microorganisms, which cause severe infections especially in immunocompromised individuals. Due to the widespread occurrence of microorganisms which cause biofilm-related infections and increased resistance of microorganisms towards the commercially available antibiotics, there is an increased interest in the discovery of novel drugs against the biofilms (1). National Institute of Health (NIH) has estimated that three out of four bacterial infections are biofilm-related. Biofilms can withstand host immune responses more than their nonattached, planktonic bacterial ones. Biofilms can cause clogging of the capillary blood vessels in the circulatory system or brain, creating plaques and causing gingivitis in the oral cavity of human or animals (2). The total annual cost of these adverse consequences of biofilms reaches billions of dollars; thus novel antimicrobials and/or new approaches to combat the problem are urgently needed (3)

*Pseudomonas aeruginosa* is not only an important opportunistic pathogen (4), but also a causative agent of emerging nosocomial infections that is considered as a model organism for the study of diverse bacterial mechanisms contributed to bacterial persistence in the environment (5). Bismuth-thiols (BTs) are a group of antibacterial agents with anti-biofilm activity against gram-positive and -negative bacteria. The thiol element functions as a lipophilic carrier that enhances bismuth uptake through bacterial surface up to 1000-fold (6). Bismuth acts as a metabolic poison inside the cell, results in growth inhibition and cell death (7). At sub inhibitory concentrations, BTs inactivate the bacterial respiratory enzymes, suppress the exopolysaccharide expression and inhibit biofilm formation in Gram-positive and -negative bacteria. Furthermore, BTs interfere with the bacterial adherence and colonization, and increase the susceptibility of bacteria to host defenses. 

Domenico et al. showed that at the sub inhibitory concentration, bismuth dimercaprol (BisBAL) inhibits capsule expression, which promotes phagocytosis and increases the reactivity of certain antibodies against lipopolysaccharide (LPS) O antigen, LPS core epitopes or outer membrane proteins (8). One approach to prevent infection caused by the indwelling devices is the coating of the catheter by antimicrobial agents. The possible advantage of BTs over other metal-based antiseptics and antifouling agents e.g. silver, copper, and organotin is their relative non-toxicity compared to the other heavy metals (9).

## 2. Objectives

The aim of the present study was to evaluate the *in vitro* anti-biofilm activity of several antibacterial combinations. For this reason, bismuth ethane dithiol (BisEDT), bismuth propane dithiol (BisPDT) as biocides and imipenem (Im), ciprofloxacin (Cp), and ceftazidime (Cz) as antibiotics were selected to test their synergistic inhibitory effects on *P. aeruginosa* biofilm formation.

## 3. Materials and Methods

*P. aeruginosa* 214, a previously isolated clinical strain, was used for the experiments, which was previously reported to form strong biofilms (10).

### 3.1. Bismuth Thiolsand Antibiotics

Two bismuth thiols, bismuth 1,2-ethaneditiol (BisEDT) and bismuth 1,3-propanedithiol (BisPDT) were used against *P. aeruginosa* 214. All thiols were purchased from Sigma Aldrich and prepared in propylene glycol by mixing bismuth nitrate and thiol in a 2:1 ratio. Cp was purchased from Temad (Iran), Cz from Qilu (China), and Im from Choongwae (Korea). All antibiotic powders were dissolved in the appropriate solvents according to the manufacturer's’ instructions. Antibiotic solutions were then sterilized using 0.22 µm filters and stored at -20˚C.

### 3.2. Antibiofilm Activity and Synergy Studies

Serial dilutions of biocides and antibiotics were prepared in Tryptic Soy Broth (TSB) plus 0.2% glucose (antibiotic concentrations from 10 to 0.156 µg/mL and biocide concentrations from 1.55 to 0.001 µM). Checkerboard arrangements of biocides and antibiotics were prepared in 96-well microtiter plates as previously described. In the checkerboard technique, two drugs are compared in microtiter wells using the drug concentrations equal to, above and below the MIC of the drugs being tested. In our study antibiotic concentrations were chosen to be from 0.078 to 40 µg/mL. For imipenem, the concentrations of 80 and 160 µg/mL were also tested. BT concentrations were chosen to be from 1.55 to 0.001 µM (11). Each microtiter plate contained five wells for sterility control samples, five wells for growth controls eight wells for different concentrations of biocides and antibiotics alone and 78 wells for different combinations of biocides and antibiotics. 

The turbidity of incubated bacterial suspension was adjusted to 0.5 McFarland’s standard to achieve 108 CFU/mL. Microtiter plates were then incubated at 37˚C for 24 hours. Semiquantitative measure of biofilm formation was performed using XTT kit purchased from Roche Company (Germany). After 24 hours of incubation, the well contents were aspirated carefully and rinsed three times with sterile PBS and fixed by drying for one hour in a 37˚C-incubator. Once the wells were fully dried, 200 µL of the XTT solution was added to the wells. The plates were covered with foil to protect XTT from light and were incubated at 37˚C for 5 hours. After the incubation, the well contents were transferred to a new plate and the optical density (OD) was measured at 450 nm using an ElISA reader (12). Fractional biofilm inhibitory concentration (FBIC) of each agent was calculated from the minimum biofilm inhibitory concentration (MBIC) as follows (Equations 1 and 2) (13).

Equation 1. $$\mathrm{FBI}C_{A}=\mathrm{MBI}C_{A(C)}/\mathrm{MBI}C_{A(A)}\mathrm{FBI}C_{B}=\mathrm{MBI}C_{B(C)}/\mathrm{M BI}C_{B(A)}$$

Equation 2. $$\sum_{}^{}\mathrm{FBIC}=\mathrm{FBI}C_{A}+\mathrm{FBI}C_{B}$$

Where subscripts A and B denote antimicrobial agents A and B, and subscripts in parentheses denote the activity measurements in combination and alone, respectively. Synergy was defined as an FBIC index of ≤ 0.5, no interaction was reported when index was between> 0.5 - 4 and antagonism was considered at > 4 (14). Each assay was repeated twice. 

## 4. Results

MBIC_A_ and MBIC_C_ values are shown in Table 1. The FBIC indices were calculated and the results were interpreted accordingly (Table 1). A multiparamater ANOVA was used and the variables were considered significant at P < 0.05. The biofilm formation of *P. aeruginosa* in the presence of Cp was remarkably affected by adding BisEDT and BisPDT. The FBICs of Cp in combination with BisEDT and BisPDT were calculated 0.02 and 0.5, respectively which showed synergism between Cp and two BTs.

**Table 1.** MBIC of Antibiotics and Biocides Alone and in Combination; FBIC Values

MBIC of Biocides and Antibiotics Alone	MBIC of Biocide and Antibiotic Combinations	FBIC
**BisEDT:0.097 µM**	BisEDT:0.097 µM & Im:40 µg/mL	BisEDT+Im = 2
**BisPDT:0.097 µM**	BisEDT:0.006 µM & Cz:10 µg/mL	BisEDT+Cz = 1.06
**Im:40 µg/mL**	BisEDT:0.001 µM & Cp:0.625 µg/mL	BisEDT+Cp = 0.02
**Cz:10 µg/mL**	BisPDT:0.001 µM & Im:0.156 µg/mL	BisPDT+Im = 0.01
**Cp:32 µg/mL**	BisPDT:0.006 µM & Cz:0.625 µg/mL	BisPDT+Cz = 0.5
	BisPDT:0.001 µM & Cp:10 µg/mL	BisPDT+Cp = 0.12

**Figure 1.**
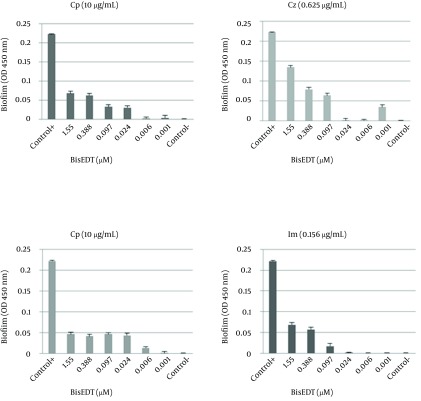
Bars Indicate Biofilms’ Biomass in the Presence of Different Concentrations of Biocides Plus Antibiotics

The FBIC of Cz in combination with BisEDT and BisPDT were 1.06 and 0.12, respectively, which shows synergistic effect between Cz and BisPDT; although no synergistic effect between BisEDT and Cz was observed. The FBIC of Im in combination with BisEDT and BisPDT were 2 and 0.01, respectively which shows no interaction between Im and BisEDT and indicates a strong synergism between Im and BisPDT. In the Figure 1, the biofilm formation at different concentrations of BisEDT and BisPDT plus antibiotics are shown. As it is obvious from the charts, by decreasing BisEDT and BisPDT concentrations, the amount of biofilm formation decreased, and more biofilm was formed at higher concentrations of two biocides. 

It seems that the biocides have stimulated biofilm formation at certain concentrations. BisEDT stimulated biofilm formation at concentrations above 0.001 µM in combination with the Cp. We also observed that BisPDT stimulated biofilm formation at the same concentrations in combination with the Cp and Im and at above 0.006 µM concentrations when used in combination with the Cz.

## 5. Discussion

Decreased permeability to antimicrobials is a major reason for high resistance of biofilms to antimicrobial agents which is caused by the biofilm exopolysaccharide (13, 15). Consequently, high dosage of antimicrobials is required to eradicate biofilms. Using antibiotics in combination with other antimicrobials as permeabilizers can lead to synergistic effects and decrease the effective dosage of antibiotics. Drug synergism occurs when drugs can interact in ways that enhance or magnify one or more desired or side effects of those drugs compared to when used alone (16). Depending on their dosage, BTs can be bactericidal or bacteriostatic against many gram-positive and -negative bacteria (17). 

Bismuth dimercaprol (BisBAL) reduces the polysaccharide production by *P. aeruginosa* ECR1 biofilms and increases its susceptibility to antimicrobial agents (18). BisEDT inhibits EPS and LPS production, reduces adherence to tissues in culture and increases the serum sensitivity of the *P. aeruginosa* PAO1. It has been reported that BisEDT can form an aggregation of electron dense material on the bacterial outer surface and induce blebbing in *P. aeruginosa*. In addition, following BisEDT treatment ribbon like structures appear around the bacterial surface that seem to contain LPS (9). It was also reported that LPS is one of the main causes of toxicity and the main site of metal binding in Gram-negative bacteria (19).

In this study three families of antibiotics were used. Ceftazidime is a third generation cephalosporin which is effective against Gram-negative bacteria especially *P. aeruginosa*. The known mechanisms of resistance to β-lactam antibiotics include the expression of β-lactamase, alteration of drug target, and reduction of bacterial permeability and increase of drug efflux. By using the BisPDT together with the Cz, MBICs of both antimicrobial agents reduced by more than 16 times indicating the synergistic effects on biofilm inhibition according to the FBIC index. Using BisEDT in combination with Cz may have disturbed efflux pumps and increased bacterial permeability which leads to an increase in intercellular concentration of Cz. According to Domenico et al. bismuth can augment the Cz effect on *P. aeruginosa* and in our study similar results were observed in the biofilm inhibition assays (7). Loss of OprD, a porin that forms narrow transmembrane channels, is an important mechanism of resistance to imipenem (20). The addition of 0.001 µM BisPDT reduced the MBIC of the Im against bacteria by 356 times. FBIC index was 0.01 which showed strong synergism between the two components. It seems that the degradation of membrane by BisPDT could reduce the resistance to imipenem.

Ohge et al. has reported that bismuth can reduce the Im efficacy on bacterial growth by up to 20 folds but in this study we observed a synergism between Im and BisPDT in our biofilm inhibition experiments (21). Ciprofloxacin is a synthetic chemotherapeutic antibiotic of the flouroquinolone class (22). Of the known mechanisms of flouroquinolone resistance, one of them is in related to efflux pumps that can decrease the intracellular quinolone concentration (23). Using Cp in combination with BisEDT and BisPDT shows synergistic effects on biofilm inhibition. The addition of 0.001 µM BisEDT reduced the MBIC of Cp by 50 folds and addition of the same amount of BisPDT reduced the Cp MBIC by 3 folds. BT inhibits the EPS and slime production and consequently inhibits biofilm formation so antibiotics can penetrate into the bacteria more easily. BTs also affect bacterial membranes and LPS; this can disturb efflux pumps and increase the intracellular concentration of antibiotics. 

According to Domenico et al. bismuth can work in synergism with Cp and enhance its antibacterial effects. We also observed increased antibiofilm activity of Cp by using it together with bismuth thiols. However the exact mechanism of this synergistic effect is currently unknown. As mentioned before, BTs can stimulate biofilm formation at certain concentrations. Shemesh and his colleagues reported that biofilm formation is enhanced by sub-lethal doses of chlorine dioxide (ClO_2_), resulted in acceleration of *Bacillus subtilis* biofilm formation as well as other bacteria, suggesting that biofilm formation is a widely conserved response among various bacterial species to sub lethal doses of this agent. They indicated that biofilm formation is a self-protective response that helps to protect the bacteria cells from the toxic effects of biocides (24). Not only the biocides, but some antibiotics like aminoglycosides can induce biofilm formation in sub-inhibitory concentrations, which is a protective response against harmful environmental agents (25). Therefore, the proper amount of biocide should be chosen very carefully. Reducing the effective dose of drugs and consequently, their side effects and toxicity are some advantages of combined antimicrobial therapy. Preventing the selection of resistant microorganisms is another advantage of combination therapy. 

Combination therapy is also useful in polymicrobial infections due to broad- spectrum coverage for the initial therapy of severely infected patients (26). BTs are effective at nontoxic low l concentrations, so they can be used as an adjunct to reduce the effective dose of antibiotics. It can be concluded that appropriate concentrations of BTs in combination with antibiotics had synergistic effects on *P. aeruginosa* biofilm inhibition. However, future investigations must be carried out on other clinically important bacteria such as *Staphylococcus* and *Acinetobacter*, which are also able to produce persistent biofilms.
